# Hsa_circ_0012563 promotes migration and invasion of esophageal squamous cell carcinoma by regulating XRCC1/EMT pathway

**DOI:** 10.1002/jcla.23308

**Published:** 2020-03-17

**Authors:** Zhuo Zhang, Xueman Li, Fei Xiong, Zhangtao Ren, Yongming Han

**Affiliations:** ^1^ Department of Thoracic Surgery Tongren Hospital of Wuhan University (Wuhan Third Hospital) Wuhan China; ^2^ Department of Pharmaceutical Sciences Tongren Hospital of Wuhan University (Wuhan Third Hospital) Wuhan China; ^3^ School of Basic Medicine Hubei University of Chinese Medicine Wuhan China

**Keywords:** EMT, ESCC, hsa_circ_0012563, invasion, migration, XRCC1

## Abstract

**Background:**

Recent reports have indicated that circular RNA (circRNA) may regulate tumorigenesis development. However, the function of circRNAs in esophageal squamous cell carcinoma (ESCC) is unclear.

**Material and method:**

The RT‐qPCR assay was performed to detect hsa_circ_0012563 expression in ESCC tissues and cell lines. Then, the MTT assay, colony formation assay, flow cytometric assay, and cell migration and invasion assay were performed to examine the function of hsa_circ_0012563. In addition, the RT‐PCR and Western blot were used to detect XRCC1 and epithelial‐to‐mesenchymal transition (EMT) related gene expression.

**Results:**

The RT‐qPCR revealed that the hsa_circ_0012563 expression was remarkably upregulated in ESCC tissue and ESCC cell lines. Functionally, downregulation of hsa_circ_0012563 suppressed cell proliferation, migration, and invasion and promoted cell apoptosis. Mechanically, the knockdown of hsa_circ_0012563 inhibited XRCC1‐mediated EMT pathway to suppress cell migration and invasion.

**Conclusions:**

Therefore, these results reveal hsa_circ_0012563 is a critical oncogene and may be a novel biomarker in ESCC.

## 1 INTRODUCTION

Esophageal cancer (EC), the 8th most common cancer in the world, is widely regarded as a genetic disease.^1^ Esophageal squamous cell carcinoma (ESCC), one of the main subtypes of EC, is a malignancy that arises from esophageal epithelial cells.^2^ However, the molecular mechanisms of ESCC remain largely unknown.^3^, ^4^

Circular RNA does not generally encode protein and can occur in any genomic region, and 85% of circular RNAs are aligned in sense orientation to known protein‐coding genes.^5^ Compared with linear RNAs, circRNA covalently linked 3′ and 5′ ends to form a closed loop6 and possess high stability, and it highly represented in the eukaryotic transcriptome.^6^, ^7^ Recently, a lot of circular RNAs have been identified in tumor and are closely related to tumor development and metastasis.^8^, ^9^, ^10^, ^11^ Notably, recent studies showed circRNAs could sponge miRNAs to suppress function of miRNA. For example, circular RNA hsa_circ_0001564 regulates osteosarcoma proliferation and apoptosis by acting miRNA sponge.^12^ CircRNA_100269 is downregulated in gastric cancer and suppresses tumor cell growth by targeting miR‐630.^13^ Hsa_circ_0000673 is downregulated in gastric cancer and inhibits the proliferation and invasion of tumor cells by targeting miR‐532‐5p.^14^ Hsa_circ_0004370 promotes esophageal cancer progression through miR‐1294/LASP1 pathway.^15^

X‐ray repair cross‐complementing group 1 (XRCC1), one of the DNA repair genes encoding a scaffolding protein, participates in base excision repair (BER) pathway.^16^ Early study indicated XRCC1 was associated with cell migration and invasion. For example, upregulation of XRCC1 suppressed tumor migration and invasion in glioma.^17^ Nevertheless, the correlation of XRCC1 and epithelial‐mesenchymal transition (EMT) in ESCC is unclear.

Recently, research has been showed ZYG11A is a potential oncogene that promotes NSCLC cell proliferation and migration in vitro and in vivo.^18^ The hsa_circ_0012563 is located at chr1:53308182‐53333449, and its associated‐gene symbol is ZYG11A. This data were from circbase (http://www.circbase.org/cgi‐bin/simplesearch.cgi). Thus, hsa_circ_0012563 was chosen for this study. Notably, biological function and mechanism of hsa_circ_0012563 in tumor are unclear. Therefore, in this study, we detected expression of hsa_circ_0012563 in ESCC and examined its biological function and mechanism in vitro.

## 2 MATERIALS AND METHODS

### 2.1 Patients and tissue samples

All 60 pairs of ESCC tissues and adjacent normal tissues were obtained at the Department of Thoracic Surgery, Tongren Hospital of Wuhan University (Wuhan Third Hospital), between 2016 and 2018. We confirmed that all methods accord to HIPAA guidelines and approved institutional protocols and written informed consent were obtained from all patients. The research was approved by the Ethics Committee of Department of Thoracic Surgery, Tongren Hospital of Wuhan University (Wuhan Third Hospital).

### 2.2 Cell culture

A normal human esophageal epithelial cell line HEEC and human ESCC cell lines TE‐13, KYSE‐410, ECA‐109, and TE‐1 cells were purchased by Type Culture Collection of Chinese Academy of Sciences (Shanghai, China). KYSE‐410 and HEEC cells were cultured in RPMI 1640 medium (Gibco; Thermo Fisher Scientific, Inc), and TE‐13, ECA‐109, and TE‐1 cells were cultured in DMEM medium (Gibco; Thermo Fisher Scientific, Inc). All medium were supplemented with penicillin‐streptomycin and 10% FBS (FBS; Gibco; Thermo Fisher Scientific, Inc), and all cells were maintained at 37℃ in an atmosphere of 5% CO_2_.

### 2.3 Cell proliferation assay

Cell proliferation was examined by using the MTT assay (MTT; SigmaAldrich) following the manufacturer's instructions (Biosharp). The cells were seeded in 96‐well plates (5000 cells/well). Cell proliferation was examined after 24 hours, 48 hours, 72 hours, and 96 hours. The solution was then measured spectrophotometrically at 490 nm.

### 2.4 Cell cycle and apoptosis assay by flow cytometric

The cell cycle was detected by labeling cells with PI for 15 minutes, and the cells were then detected by using a FACScan analyzer. The transfected cells were harvested after 48 hours and were then stained with Annexin V and PI (BD Pharmingen) before analysis by flow cytometry (C6; BD Biosciences).

### 2.5 Clonogenic assay

The 1000 cells were plated in 6‐well plate. After 7 days, the cells were immobilized with 4% paraformaldehyde and stained with crystal violet. The visible colonies were then manually counted with ImageJ.

### 2.6 Cell migration assays

For migration assays, cells (500 000 cells/100 µL per well) were plated in the upper chamber (8 mm pores, Millipore) with non‐FBS DMEM, and the lower chamber was filled with medium containing 10% FBS. The cells were incubated for 24 hours, and the migration on the lower membrane surface was assessed by staining with Hoechest 33 342 and counted under a microscope.

### 2.7 Cell invasion assays

For invasion assays, cells (500 000 cells/100 mL per well) were plated in the upper chamber (8 mm pores, Millipore) with a matrigel‐coated membrane in non‐FBS DMEM, and the lower chamber was filled with medium containing 10% FBS. The cells were incubated for 24 hours, and the invasion on the lower membrane surface was assessed by staining with Hoechest 33 342 and counted under a microscope.

### 2.8 Real‐time quantitative PCR

Total RNA was extracted by using TRIzol reagent (Invitrogen). For RT‐qPCR analysis, cDNA was reverse transcribed by Reverse Transcription Kit (Toyobo). RT‐qPCR was performed with SYBR Green kit (Qiagen). All sample were run by the ABI Prism 7500 Sequence Detector (Applied Biosystems). The mRNA expression level was calculated and normalized by using the 2^‐ΔΔCt^ method relative to GAPDH. The primers for hsa_circ_0012563, E‐cadherin, N‐cadherin, Vimentin, snail, and GAPDH were showed in Table 1.

**Table 1:** The primer sequences included in this study

Name	Primer sequences (5′‐3′)
hsa_circ_0012563	
Forward	CTACGTTTGTTACCGTCACTC
Reverse	GAGGGGTCGGAGGGGTCGGAG
GAPDH	
Forward	GCACCGTCAAGGCTGAGAAC
Reverse	ATGGTGGTGAAGACGCCAGT

### 2.9 Western blot

Total protein was collected by using RIPA lysis buffer (Beyotime), and protein concentrations were examined with the bicinchoninic acid method (BCA, Beyotime). The proteins were used for 12% SDS‐polyacrylamide gels and transferring onto PVDF membranes. Primary antibodies against XRCC1, E‐cadherin, N‐cadherin, and GAPDH were obtained from Abcam. All the secondary antibodies were purchased from Cell signaling technology.

### 2.10 Statistical analysis

Differences between groups were assessed by using paired sample *t* test, and all data are represented by the mean ± SD. A *P*‐value of <.05 (*) was considered statistically significant; A *P*‐value of <.01(**) was considered statistically very significant; A *P*‐value of <.001(***) was considered statistically very much significant.

## 3 RESULT

### 3.1 Hsa_circ_0012563 is upregulated in both clinical specimens and ESCC cell lines

**Figure 1:**
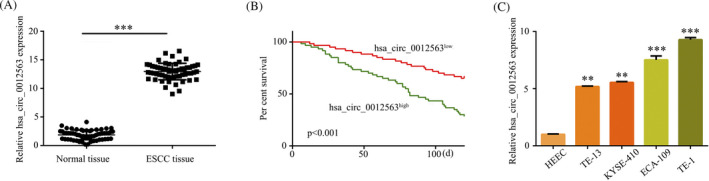
Hsa_circ_0012563 is upregulated in both clinical specimens and ESCC cell lines. (A) Hsa_circ_0012563 was significantly increased in ESCC tissues compared with normal tissues. (B) Kaplan‐Meier survival analysis. (C) Hsa_circ_0012563 expression was examined via RT‐qPCR in human esophageal epithelial cell line HEEC and human ESCC cell lines TE‐13, KYSE‐410, ECA‐109, and TE‐1 cells. The data shown represent the mean ± SD (n = 3). *P < .05, **P < .01, ***P < .001. GAPDH was used as the internal control

The expression level of hsa_circ_0012563 in ECSS tissue and normal tissue was initially evaluated by RT‐qPCR. The result showed that the expression level of hsa_circ_0012563 was significantly increased in ESCC compared with normal tissues (Figure 1A). Higher hsa_circ_0012563 expression in ESCC patients was related to lower survival rate by Kaplan‐Meier survival analysis (Figure 1B). It yet showed hsa_circ_0012563 expression was significantly increased in ECSS cell lines compared with HEEC cells (Figure 1C). Therefore, these results demonstrate hsa_circ_0012563 is consistently expressed in both ESCC tumor tissues and ESCC cell.

### 3.2 Hsa_circ_0012563 exerts a tumor‐promotive function in ESCC

**Figure 2:**
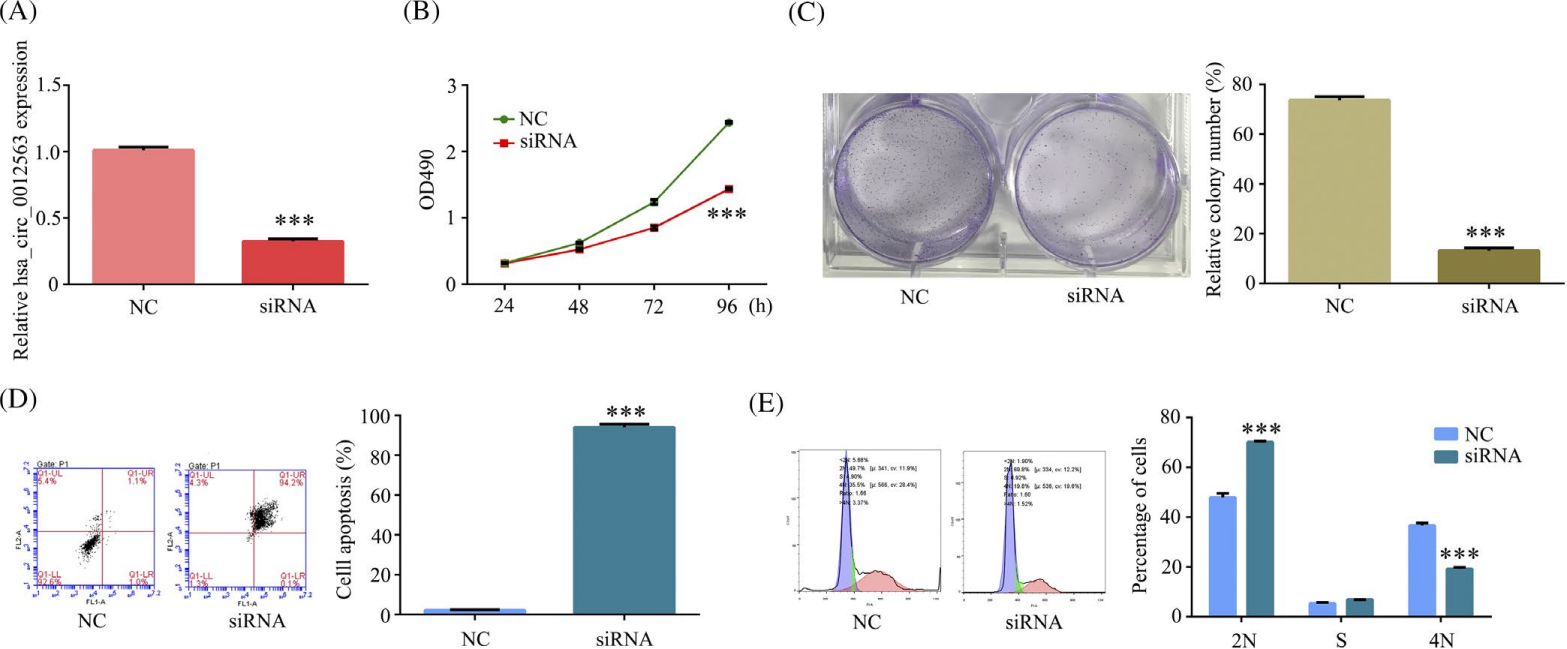
Hsa_circ_0012563 exerts a tumor‐promotive function in ESCC. (A) Hsa_circ_0012563 expression was suppressed by transfected siRNAs in ESCC cells. The effect of hsa_circ_0012563 on cell proliferation was detected by using MTT (B) and colony formation (C) assays. Flow cytometry was performed to detect the cell apoptosis (D) and cell cycle phase (E) in TE‐1 cell‐si‐hsa_circ_0012563. All the data shown represent the mean ± SD (n = 3). *P < .05, **P < .01, ***P < .001. All siRNA was si‐hsa_circ_0012563

To explore hsa_circ_0012563 exerts a tumor‐promotive function in ESCC, we designed siRNA to suppress hsa_circ_0012563 expression. RT‐qPCR showed hsa_circ_0012563 expression was inhibited in transfected si‐hsa_circ_0012563 TE‐1 cell compared with si‐NC TE‐1 cell (Figure 2A). In addition, the cell proliferation was significantly inhibited in TE‐1 cell‐si‐hsa_circ_0012563 at 48 hours, 72 hours, and 96 hours, but no significant difference at 24 hours (Figure 2B), and the inhibition of hsa_circ_0012563 expression markedly weakened clone formation ability of TE‐1 cell (Figure 2C), explained that hsa_circ_0012563 promoted ESCC proliferation in vitro. Then, the flow cytometric analyses were performed to further detect whether hsa_circ_0012563 affects ESCC cell proliferation via altering cell apoptosis or cycle progression. The results revealed that si‐hsa_circ_0012563 transfection promoted ESCC cell apoptosis (Figure 2D) and induced G1 arrest (Figure 2E) compared with si‐NC transfection. Therefore, hsa_circ_0012563 exerts a tumor‐promotive function in ESCC.

### 3.3 Downregulated of hsa_circ_0012563 inhibited TE‐1 cell migration and invasion in vitro

**Figure 3:**
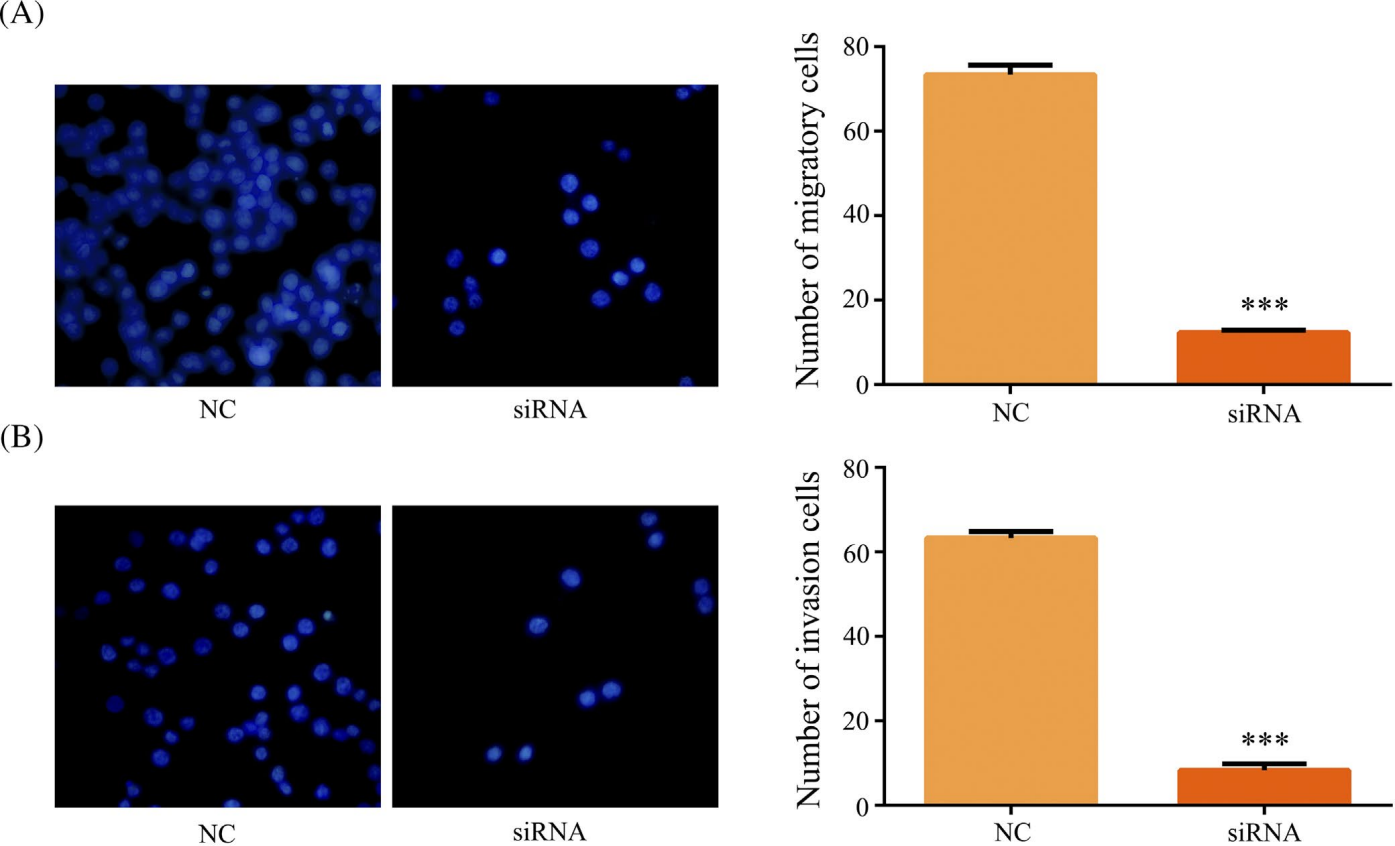
Downregulated of hsa_circ_0012563 inhibited TE‐1 cell migration and invasion in vitro. Knockdown of hsa_circ_0012563 inhibited cell migration (A) and invasion (B) in TE‐1 cells. All data shown represent the mean ± SD (n = 3). *P < .05, **P < .01, ***P < .001. All siRNA was si‐hsa_circ_0012563

As shown in Table 2, hsa_circ_0012563 expression was associated with metastasis and suggested that hsa_circ_0012563 may promote ESCC metastasis. In addition, the migration assays revealed knockdown of hsa_circ_0012563 suppressed migratory ability of TE‐1 cells (Figure 3A). Invasion assay also showed that hsa_circ_0012563 knockdown remarkably inhibited the invasive capacity of TE‐1 cells (Figure 3B). Therefore, overexpression of hsa_circ_0012563 promotes ESCC cell migration and invasion in vitro.

**Table 2:** Relationship between clinical features and hsa_circ_0012563 expression in 60 ESCC patients

No. of Variables	Cases	hsa_circ_0012563 expression		*P*‐value
		Low (n %)	High (n %)	
Age (years)				
<55	32	12	20	.525
≥55	28	12	15	
Gender				
Male	31	12	19	.833
Female	29	12	17	
TNM stage				
I‐II	22	7	15	.325
III‐IV	38	17	21	
Tumor size				
<5 cm	20	12	8	.025
≥5 cm	40	12	28	
Metastasis				
No	21	18	3	<.05
Yes	39	6	33	

### 3.4 Hsa_circ_0012563 promoted cell migration and invasion by regulating XRCC1/EMT pathway

**Figure 4:**
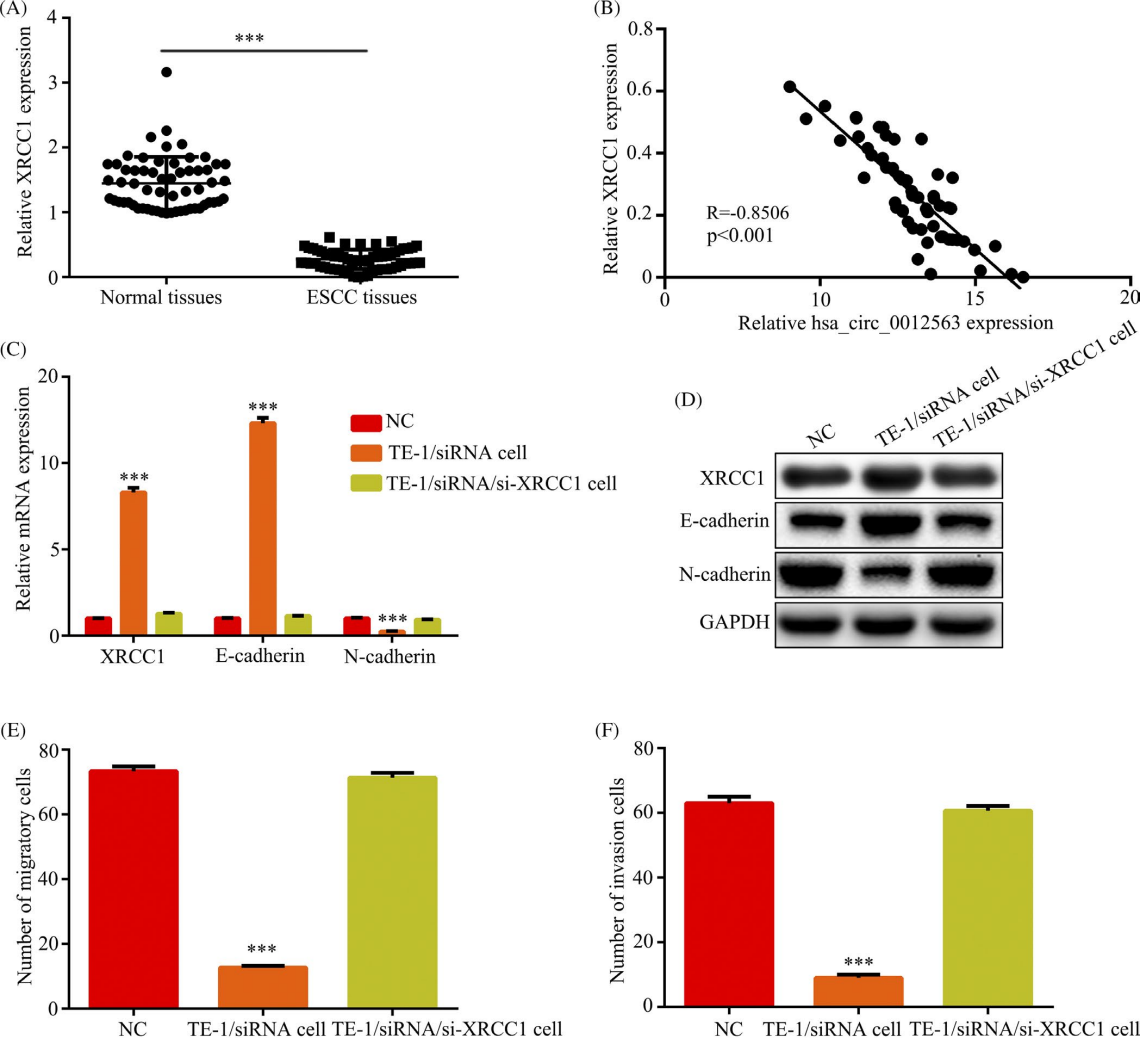
Hsa_circ_0012563 promoted cell migration and invasion by regulating XRCC1/EMT pathway. (A) XRCC1 expression was significantly increased in ESCC tissues compared with normal tissues. (B) The correlation between hsa_circ_0012563 and XRCC1 expressions in 60 ESCC patients was evaluated by using Spearman's correlation analysis (n = 60, *r* = −.8506, *P* < .01). Expression levels of XRCC1 and EMT‐related protein were examined by RT‐qPCR (C) and Western blot (D) in TE‐1 cell‐si‐hsa_circ_0012563 and TE‐1 cell‐si‐hsa_circ_0012563‐si‐XRCC1. Knockdown of XRCC1 promoted cell migration (E) and invasion (F) in TE‐1 cell‐si‐hsa_circ_0012563. All data shown represent the mean ± SD (n = 3). *P < .05, **P < .01, ***P < .001. All siRNA was si‐hsa_circ_0012563

Our data have revealed that hsa_circ_0012563 was related to migration and invasion of ESCC, and we further explored metastasis mechanism. Then, early reports pointed out that XRCC1 was associated with cell migration and invasion.^17^ Thus, we evaluated expression levels of XRCC1 by RT‐qPCR assay in 60 ESCC patients. The result showed that the XRCC1 expression levels were significantly decreased in tumor tissues compared with the corresponding adjacent normal tissues (Figure 4A). The XRCC1 expression was inversely correlated with hsa_circ_0012563 in 60 ESCC patients by Pearson's correlation analysis (Figure 4B). The RT‐qPCR and Western blot indicated that knockdown of hsa_circ_0012563 could promote expression of XRCC1, E‐cadherin and inhibit N‐cadherin expression, and downregulation of XRCC1 restores E‐cadherin and N‐cadherin expression of transfected si‐hsa_circ_0012563 TE‐1 cell (Figure 4C and D), suggested that hsa_circ_0012563 activated EMT pathway by inhibiting XRCC1 expression in ESCC cell. In addition, the migration and invasion assay revealed that downregulation of XRCC1 restores migration and invasion of transfected si‐hsa_circ_0012563 TE‐1 cell (Figure 4E and F). Thus, hsa_circ_0012563 promoted cell migration and invasion by regulating XRCC1/EMT pathway.

## 4 DISCUSSION

Esophageal cancer is a complex, dynamic, and biological processes, and multiple genes are related to ESCC.^19^ In addition, metastatic relapse is one of the main causes of poor prognosis of ESCC, including cell adhesion, migration, and reaching target organs, and the lncRNAs and miRNAs are also related to pathogenesis of ESCC. However, it is not currently clear in the literature whether circRNAs are associated with ESCC. Thus, it is significant to explore the molecular mechanisms of carcinogenesis and progression in ESCC to develop novel strategies to improve ESCC prognosis.

Recently, many studies pointed that circRNAs are related to tumor pathogenesis and metastasis. For example, ZKSCAN1 gene and its related circular RNA (circZKSCAN1) both inhibit hepatocellular carcinoma cell growth, migration, and invasion.^20^ Downregulated hsa_circ_0001649 regulates proliferation, migration, and invasion in cholangiocarcinoma cells.^21^ circDDX17 acts as a tumor suppressor in colorectal cancer.^22^ Notably, circRNAs as an excellent molecular biomarker plays an indispensable role in tumor. For instance, hsa_circ_0001649 is a potential novel biomarker for hepatocellular carcinoma.^8^ Circular RNA circ‐LDLRAD3 is a biomarker in diagnosis of pancreatic cancer.^23^ Hsa_circ_0013958 is a potential novel biomarker for lung adenocarcinoma.^24^ In addition, circRNAs could sponge miRNAs to suppress function of miRNA. For example, circular RNA FOXO3 inhibits tumor progression in non‐small cell lung cancer through sponging miR‐155.^25^ CircRNA_100269 is downregulated in gastric cancer and suppresses tumor cell growth by targeting miR‐630.^13^ Hsa_circRNA_0006528 as a competing endogenous RNA promotes human breast cancer progression by sponging miR‐7‐5p and activating the MAPK/ERK signaling pathway.^26^ Therefore, it is important to study the function of circRNAs in tumors.

In this study, we identified a new circRNA hsa_circ_0012563 and demonstrated this circRNA is associated with ESCC development and metastasis. Hsa_circ_0012563 was firstly reported in ESCC, and hsa_circ_0012563 was remarkably upregulated in ESCC tissues and cell lines. Moreover, knockdown of hsa_circ_0012563 inhibited the progress of cell proliferation, migration, and invasion in ESCC, and knockdown of hsa_circ_0012563 promoted cell apoptosis in ESCC. Thus, these data indicated that hsa_circ_0012563 promotes progression, and migration and invasion in ESCC.

Early reports pointed out that XRCC1 was associated with cell migration and invasion.^17^ In this study, we indicated knockdown of hsa_circ_0012563 expression promoted the expression of XRCC1 and E‐cadherin in ESCC and remarkably inhibited N‐cadherin expression in ESCC. In addition, downregulation of XRCC1 restores E‐cadherin and N‐cadherin expression of transfected si‐hsa_circ_0012563 TE‐1 cell. Thus, hsa_circ_0012563 promotes migration and invasion of ESCC via regulating XRCC1/EMT pathway.

In summary, hsa_circ_0012563 is upregulated in ESCC tumor tissues and cells, and its expression is associated to tumor pathogenesis and metastatic. Further, hsa_circ_0012563 promotes migration and invasion of ESCC via regulating XRCC1/EMT pathway. Therefore, this suggests hsa_circ_0012563 is an oncogene in ESCC and may represent a novel diagnostic and therapeutic target for treatment of ESCC.

## CONFLICT OF INTEREST

The authors declare no conflict of interest.
